# Sex rearing in individuals with 46,XY disorders of sex development prior to diagnosis

**DOI:** 10.1186/1687-9856-2013-S1-P192

**Published:** 2013-10-03

**Authors:** Nanis S Marzuki, Lita P Suciati, Chrysantine Paramayuda, Hannie D Kartapradja, Bambang Tridaja

**Affiliations:** 1 Eijkman Institute for Molecular Biology, Jakarta, Indonesia; 2 Pediatric Endocrinology Division, Department of Child Health, Faculty of Medicine-Cipto Mangunkusumo Hospital, University of Indonesia

## 

46,XY Disorders of Sex Development (DSD) has wide variations of clinical manifestations ranging from complete female, genitalia ambiguity, to complete male. Therefore the inviduals with this disorder can be reared as boys or girls. Most of Indonesian DSD cases are facing with budget constraint, because they require series of costly examinations and treatment.

We aimed to know the distribution of sex rearing in inviduals with 46,XY DSD and tried to find out the possible etiologies based on the clinical manifestations and the age of presentation.

The study reported data of types of sex rearing, age at and clinical presentation of subjects obtained from 46,XY resulted chromosome analysis patients referred to our clinic in year 2009-2010.

Seventy 46,XY DSD cases (aged 1 day-32 years old) were collected and analyzed, and 45 of them were reared as boys, 22 as girls, while the types of sex rearing was not yet assigned in 3 cases. Most of the cases were referred for chromosome analysis at age > 3 months-8 years, and 12 patients at 18 years or above, 10 of them were raised as female. Genitalia ambiguity was the most frequent referral reason (44/70 cases). Based on detailed clinical examinations and age of presentation we also described the possible diagnosis, in order to help parents or patients to assign gender with minimum cost.

